# F243. INFLUENCE OF METACOGNITION AND IRRATIONAL BELIEFS ON SOCIAL FUNCTIONING IN PSYCHOSIS OF RECENT ONSET

**DOI:** 10.1093/schbul/sby017.774

**Published:** 2018-04-01

**Authors:** Helena García-Mieres, Raquel López-Carrilero, Jordi Cid, Esther Pousa, Ana Barajas, Eva Grasa, Maria Luisa Barrigon, Isabel Ruiz-Delgado, Ana de Apraiz, Fermín González-Higueras, Esther Lorente Rovira, Spanish Metacognition Study Group, Susana Ochoa

**Affiliations:** 1 Universitat de Barcelona; 2 Parc Sanitari Sant Joan de Deu; 3 Salut Mental Girona; 4 Institut de Neuropsiquiatria i Addiccions, Hospital del Mar;; 5 Centro de Higiene Mental Les Corts, Research Unit; 6 IIB-Hospital Santa Creu I Sant Pau; 7 Fundación Jiménez Díaz Hospital; 8 Servicio Andaluz Salud Málaga; 9 Servicio Andaluz de Salud; 10 ADIRM

## Abstract

**Background:**

Social functioning is affected in early psychosis stages. This affection has multiple domains, such as vocational functioning or performance of independent living skills. These different domains are also linked; so elucidating differential or generalized determinants on specific areas and global outcomes is thus a critical step in case conceptualization and the development planning of effective early interventions. The aim of this study was to test the influence of specific domains of metacognition in different and global areas of social functioning.

**Methods:**

A cross-sectional study was performed based on baseline data from a main multicenter clinical trial. The sample was composed of 122 patients with psychosis of recent onset treated at one of the nine participating mental health centers from diverse regions of Spain. The order of assessment was a sociodemographic questionnaire, the Positive and Negative Syndrome Scale (PANSS), the Social Functioning Scale (SFS), the Hinting Task (Theory of Mind, ToM), the Beck Cognitive Insight Scale (BCIS), the Internal, Personal and Situational Attributions Questionnaire (IPSAQ), the Irrational Belief Test (TCI) and the Emotional Recognition Test Faces. Pearson correlations and multiple regression analysis were performed.

**Results:**

In the first models, results showed that social engagement/withdrawal was explained by Helplessness (9.2% of the variance). Interpersonal communication was explained by Emotional Irresponsibility, internal attribution of negative events, affective JTC and emotion recognition (17.5% of variance). Independence-competence was explained by Helplessness, Emotional Irresponsibility and ToM (16% of variance). Independence-performance was explained by Helplessness (8.2% of variance). Employment/occupation was explained Emotional Irresponsibility (12.4% of variance). Prosocial Activities was explained by Helplessness and Emotional Irresponsibility (14.4% of variance). Finally, the total score of the SFS was explained by Helplessness and self-reflectiveness (16% of variance). Subsequently, in a second analysis, negative symptoms emerged as a significant mediator for most domains of social functioning.

**Discussion:**

In our results, two kind of irrational beliefs, one of the main axes of cognitive therapy, emerged as relevant for social functioning in psychosis of recent onset. However, classic social cognition and metacognition measures were less significant, only ToM and self-reflecteness influenced some aspects of social functioning. Further analysis of determinants of social functioning in psychosis should explore the role of irrational beliefs and consider them for treatment strategy, along social cognition and negative symptoms.

